# Review time in peer review: quantitative analysis and modelling of editorial workflows

**DOI:** 10.1007/s11192-016-1871-z

**Published:** 2016-02-09

**Authors:** Maciej J. Mrowinski, Agata Fronczak, Piotr Fronczak, Olgica Nedic, Marcel Ausloos

**Affiliations:** Faculty of Physics, Warsaw University of Technology, Koszykowa 75, 00-662 Warsaw, Poland; Institute for the Application of Nuclear Energy (INEP), University of Belgrade, Banatska 31b, Belgrade-Zemun, Serbia; School of Management, University of Leicester, University Road, Leicester, LE1 7RH UK; eHumanities Group, Royal Netherlands Academy of Arts and Sciences (NKVA), Joan Muyskenweg 25, 1096 CJ Amsterdam, The Netherlands; Group of Researchers for Applications of Physics in Economy and Sociology (GRAPES), Rue de la Belle Jardiniere 483, 4031 Angleur, Belgium

**Keywords:** Peer review, Editorial process, Weighted directed graph

## Abstract

In this paper, we undertake a data-driven theoretical investigation of editorial workflows. We analyse a dataset containing information about 58 papers submitted to the Biochemistry and Biotechnology section of the Journal of the Serbian Chemical Society. We separate the peer review process into stages that each paper has to go through and introduce the notion of completion rate - the probability that an invitation sent to a potential reviewer will result in a finished review. Using empirical transition probabilities and probability distributions of the duration of each stage we create a directed weighted network, the analysis of which allows us to obtain the theoretical probability distributions of review time for different classes of reviewers. These theoretical distributions underlie our numerical simulations of different editorial strategies. Through these simulations, we test the impact of some modifications of the editorial policy on the efficiency of the whole review process. We discover that the distribution of review time is similar for all classes of reviewers, and that the completion rate of reviewers known personally by the editor is very high, which means that they are much more likely to answer the invitation and finish the review than other reviewers. Thus, the completion rate is the key factor that determines the efficiency of each editorial policy. Our results may be of great importance for editors and act as a guide in determining the optimal number of reviewers.

## Introduction

Despite a variety of criticisms of its effectiveness (Wager and Jefferson 2001; Cooper 2009), peer review is a fundamental mechanism for validating the quality of the research that is published in today’s scientific literature (Baker 2002; Ware and Monkman 2008; Mulligan et al. 2013; Wareand Mabe 2015; Nicholas et al. 2015). It is a complex, multi-phase process that seems to be largely understudied (Squazzoni and Takács 2011) and there appear to be some growing concerns regarding how to improve its functioning. Given the increasing number of submitted articles and the limited pool of reviewers, acquiring a good and timely review is becoming progressively more challenging. Several journals emphasize the rapidity of their review process in order to attract submissions. Reviews can take even a year, depending on the complexity of the topic, the number of reviewers involved, and the details of the editorial procedures. In contrast, sometimes reviews can be very quick, for example when the paper is rejected directly by the editor.

In face of these problems, many suggestions have been proposed to make the peer review and editorial process more efficient and equitable (Bornmann 2011). In particular, the role of editors in the process of selecting and managing reviewers has been increasingly discussed (Schwartz and Zamboanga 2009; Kravitz et al. 2010; Newton 2010). However, these discussions are mainly focused on quality, ethical issues or qualitative recommendations for editors or reviewers (Cawley 2011; Resnik et al. 2008; Hames 2013; Wager 2006; Kovanis et al. 2015) that do not lead to measurable improvements to the efficiency of peer review process as viewed from the perspective of editors. Do the editors send out a sufficient number of reviewer invitations to obtain two or three timely reviews of a manuscript? How often should they draw on expertise of the same reviewers consuming their time and energy? How long should they wait for a review before they can repeat the invitation or assume that a response is unlikely? What is the statistical chance that reviewers will respond? Does it depend on whether they were previously reviewers for the same journal? Although it is likely that editors try to answer these and other questions when they optimise their workflow, they have to do it on their own using trial and error. Without an intensive discussion that could help to answer the aforementioned questions in a more systematic way one can be sure that the submission-publication editorial lags will be increasing in the years to come.

Our paper is meant to fill this gap with the help of quantitative analysis. We examine selected aspects of peer review and suggest possible improvements. To this end, we analyse a dataset containing information about 58 papers submitted to the Biochemistry and Biotechnology section of the Journal of the Serbian Chemical Society (JSCS). After separating the peer review process into stages that each review has to go through, we use a weighted directed graph to describe it in a probabilistic manner under the weak assumption that the process is Markovian. We test the impact of some modifications of the editorial policy on the efficiency of the whole process. Our quantitative findings allow us to provide editors with practical suggestions for improving their workflow.

The paper is organized as follows:

"Review process and initial data analysis" section describes the dataset used in the paper as well as the methodology employed to analyse the data. "Review time" section is devoted to the data driven theoretical analysis of review time. Simulations of various editorial policy scenarios and their impact on the efficiency of the process are presented in "Simulations of the review process" section. In "Discussion with conclusion" section we give concluding remarks and point out open problems that may be researched within the presented methodology in the future.

## Review process and initial data analysis

The sample we studied contains information about reviews of 58 manuscripts submitted electronically to one of the sub-editors of JSCS between November 2011 and July 2014. Each of 323 members of the sample corresponds to an invitation sent to a single reviewer and comprises the group the reviewer belongs to, the ID of the reviewed manuscript and dates associated with phases of the review process. Reviewers were divided into two groups—65 **known** reviewers are known personally by the sub-editor while 258 **other** reviewers were chosen through various different means (e.g. picked up from SCOPUS database as experts in the topic of the submitted manuscript). Reviews in JSCS are single-blind, meaning that reviewers know the names of the authors but remain anonymous themselves.

It is worth noting that out of 65 aforementioned **known** reviewers 34 were actually unique. The remaining 31 invitations were sent to a group of 13 reviewers, 9 of whom were asked to review 2 manuscripts, 3 to review 3 manuscripts, and 1 to review 4 manuscripts. Reviewers who are invited to review multiple manuscripts within a short period of time may suffer from burnout (Arns 2014). In our case, non-unique reviewers received each new invitation after 345 days on average. While this sample is not large enough to make a definite statement, we did not observe any relation between subsequent review times for non-unique reviewers. All **other** reviewers were unique.

The review process itself is separable into distinct phases that mirror interactions between the sub-editor, authors and reviewers. It begins with the invitation phase, when the sub-editor, after receiving a new submission, sends out invitations to a number of reviewers (5 on average in the JSCS case: 1 **known** and 4 **other**) and waits for their responses. If any of the invited reviewers does not respond, then after about 7 days an inquiry is sent which begins the inquiry phase. If that inquiry also remains without an answer for 10 days, then the review process for that particular reviewer ends at the no response phase and is considered finished with a negative outcome. After receiving the initial invitation or the inquiry, reviewers who do answer either confirm their willingness to write the review, which begins the confirmation phase, or reject the invitation. In the latter case, much like for reviewers who did not answer at all, the review process ends at the rejection phase and is considered finished with a negative outcome. In the former, the JSCS sub-editor waits for the report for 25 days before beginning the second possible inquiry phase by sending an inquiry. This may result in either the reviewer finishing the review and sending the report—which ends the process at the report phase and is the only outcome that is considered positive—or a lack of answer, which ends the process at the no response phase. To sum it up, there are three possible outcomes of the review process—report, no response or rejection.

**Fig. 1:**
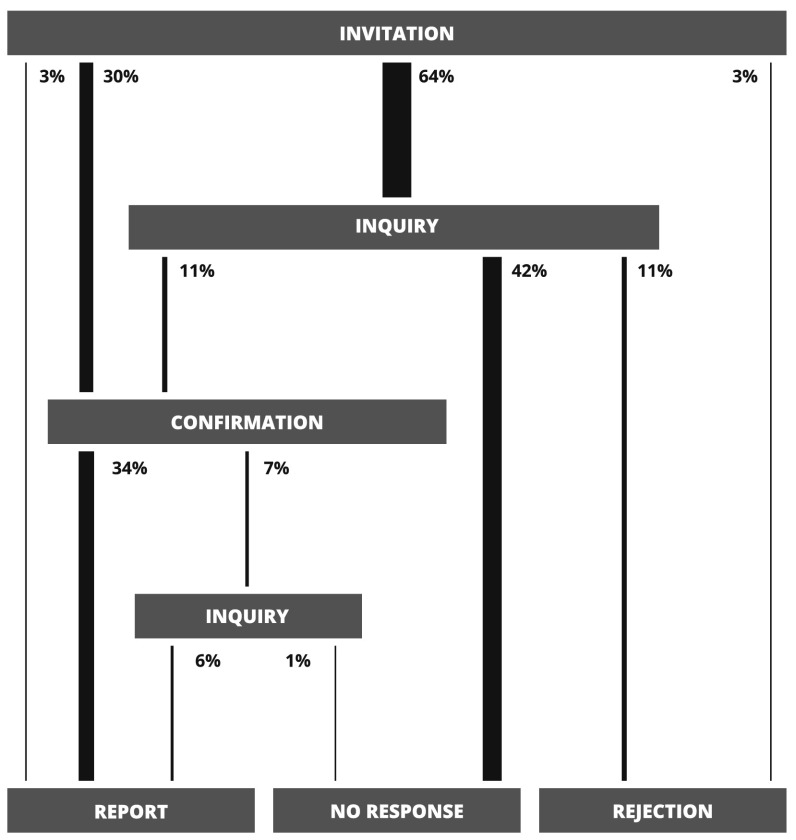
A graph corresponding to the review process with **known** and **other** reviewers. Next to each edge are probabilities (calculated as explained in the main text) of a realisation of this process passing through the edge. Transitions are only possible from the upper sections of the graph to the lower sections

**Fig. 2:**
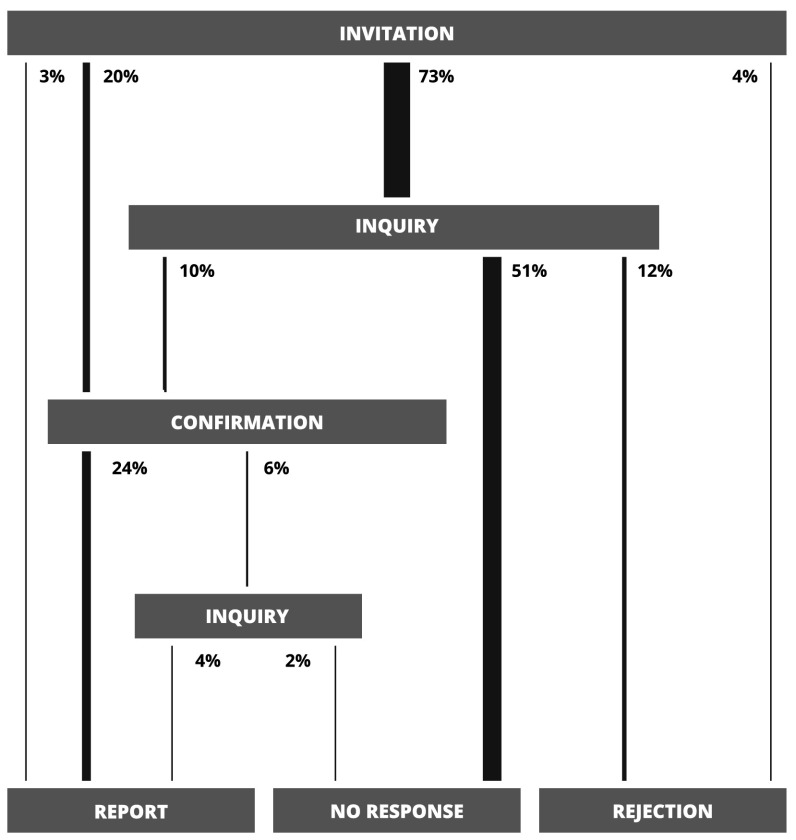
A graph corresponding to the review process with only **other** reviewers. Next to each edge are probabilities (calculated as explained in the main text) of a realisation of this process passing through the edge. Transitions are only possible from the upper sections of the graph to the lower sections

**Fig. 3:**
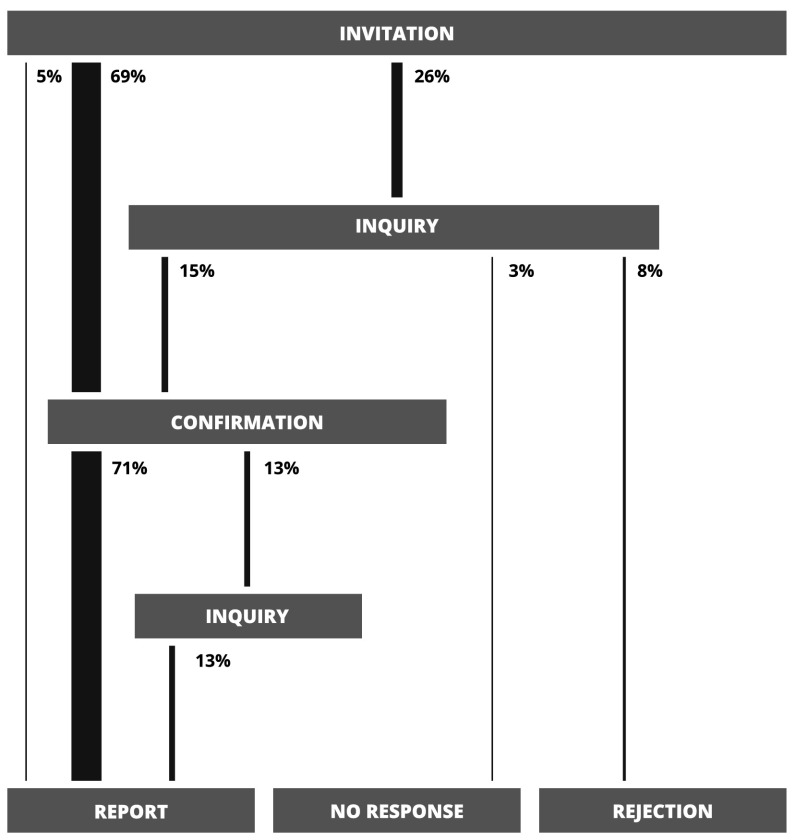
A graph corresponding to the review process with only **known** reviewers. Next to each edge are probabilities (calculated as explained in the main text) of a realisation of this process passing through the edge. Transitions are only possible from the upper sections of the graph to the lower sections

A directed graph in which nodes correspond to phases and edges to allowable transitions between subsequent phases can be used as a visual representation of the review process. Graphs that describe the workflow in our sample can be found in Figs. 1, 2 and 3. The value expressed in percent next to each edge is the probability that a realisation of the review process will pass through the edge—that is, the number of members from our sample for which the transition between nodes connected by the edge occurred divided by the size of the sample. Widths of edges were scaled proportionally to that probability.

What is immediately striking is that only 43 % of all invitations actually result in a finished review (Fig. 1). Most of reviewers—that is 64 %—do not even respond to the initial invitation and 42 % ignore the inquiry. These poor results are mostly driven by reviewers that belong to the **other** group (Fig. 2), which constitutes the majority of all reviewers. Only 31 % of **other** reviewers finish the review, 73 % ignore the initial inquiry, 51 % do not answer at all and 16 % reject the invitation. On the other hand, **known** reviewers—who are in minority—are far more reliable (Fig. 3). Most of them, 74 %, respond to the invitation and 89 % finish the review. Only 3 % do not answer and 8 % reject. As we will show in the following sections, this disparity between **known** and **other** reviewers may play a crucial role in the review process and is the key factor that determines its effectiveness.

## Review time

Review time, that is the number of days between the **invitation** phase and **report** phase, is one of the most direct and tangible measure of the efficiency of the review process. Since our sample contains information about the beginning and end of each phase, we were able to acquire distributions of review time for **known** and **other** reviewers, as well as partial distributions of days between all intermediate phases. These partial distributions are especially interesting, as they can serve as building blocks with which one can create a simulation of the entire review process and recreate the cumulative distribution of review time under various assumptions.

**Fig. 4:**
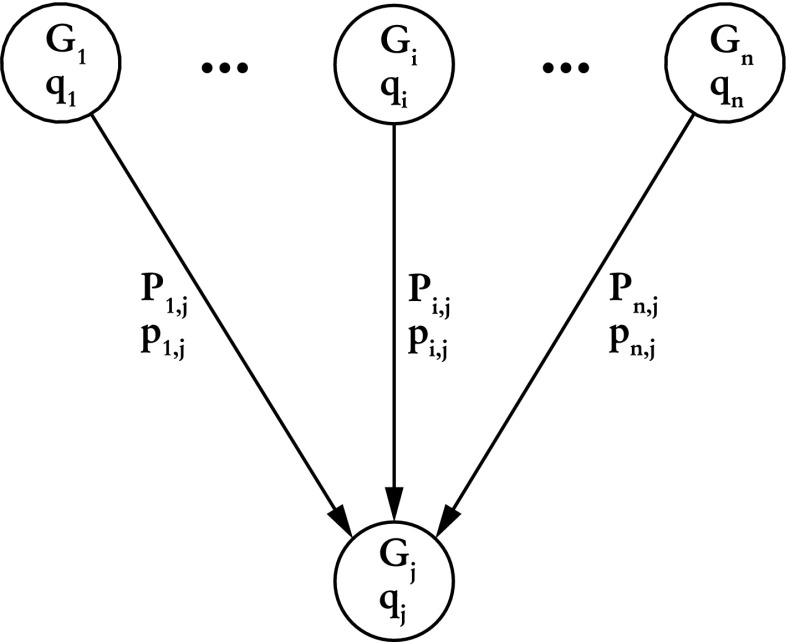
A schematic representation of a node from the review process graph, its predecessors and all associated probabilities. Detailed description can be found in "Review time" section

The distribution of review time can be reassembled using partial distributions in the following way (see Fig. 4). To each node (phase) *j* of the review process graph (Figs. 1, 2 and 3) one can assign the probability $q_j$ that a realisation of the process will pass through node *j* and the probability distribution $G_j(t)$ of days between the **invitation** phase and phase *j*. Similarly, each edge is characterised by the probability $p_{i,j}$ that the review process will pass from phase *i* to *j* and the probability distribution $P_{i,j}(t)$ of days associated with such a transition. Given all these probabilities, $G_j(t)$ can be calculated as follows

1 $$\begin{aligned} G_j(t) = \sum _{\{i\}_j} w_{i, j}\ (G_{i}*P_{i, j})(t) \end{aligned}$$

where the summation is over set $\{i\}_j$ of all predecessors of node *j* and symbol $*$ represents the discrete convolution

2 $$\begin{aligned} (G_{i}*P_{i, j})(t) = \sum _{t'=0}^{t} G_{i}(t') P_{i, j}(t-t'). \end{aligned}$$

Weights $w_{i, j}$ are defined as

3 $$\begin{aligned} w_{i, j} = \frac{q_i p_{i,j}}{q_j}. \end{aligned}$$

and the probability $q_j$ can be expressed as

4 $$\begin{aligned} q_j = \sum _{\{i\}_j} q_{i} p_{i, j}. \end{aligned}$$

Equations (1–4) are recursive. The distribution $G_j(t)$ associated with node *j* depends on the corresponding distributions associated with predecessors of node *j* and probabilities $q_j$ exhibit similar dependence. As such, these equations can be solved recursively if one assumes appropriate initial conditions for nodes without parents (in our case it is $q_{\text {invitation}} = 1$ and $G_{\text {invitation}}(t)=\delta _{0,t}$ for the node that corresponds to the **invitation** phase) and acquires probabilities $P_{i, j}$ and $p_{i,j}$ from the sample. One last fact worth noting is that the quantity $q_i p_{i,j}$ from the numerator in Eq. (3) is actually the same as the probability in Figs. 1, 2 and 3 next to each edge.

**Fig. 5:**
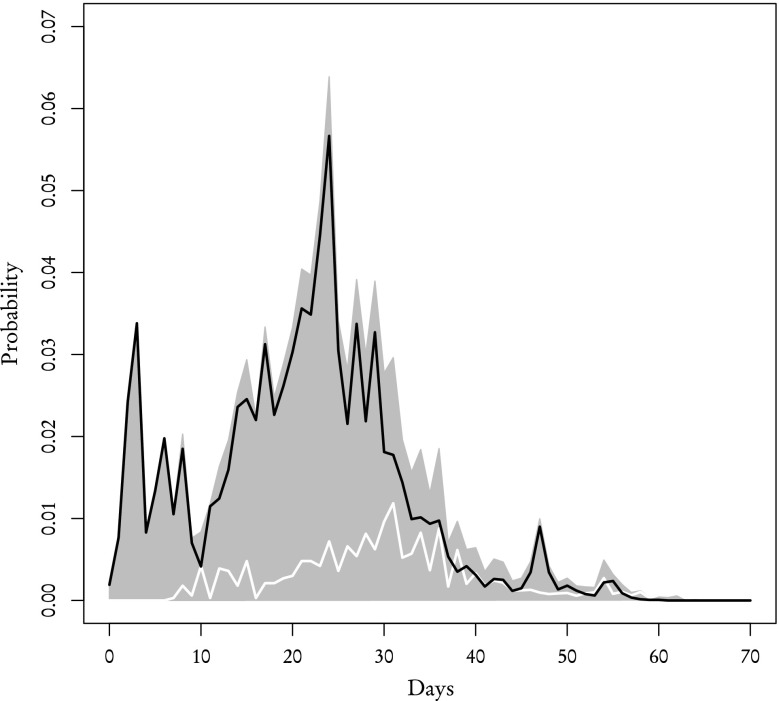
The theoretical probability distribution of review time for **known** reviewers who responded to the initial invitation (*black line*), who received an inquiry (*white line*) and their sum which gives the distribution for all **known** reviewers (*filled polygon*)

**Fig. 6:**
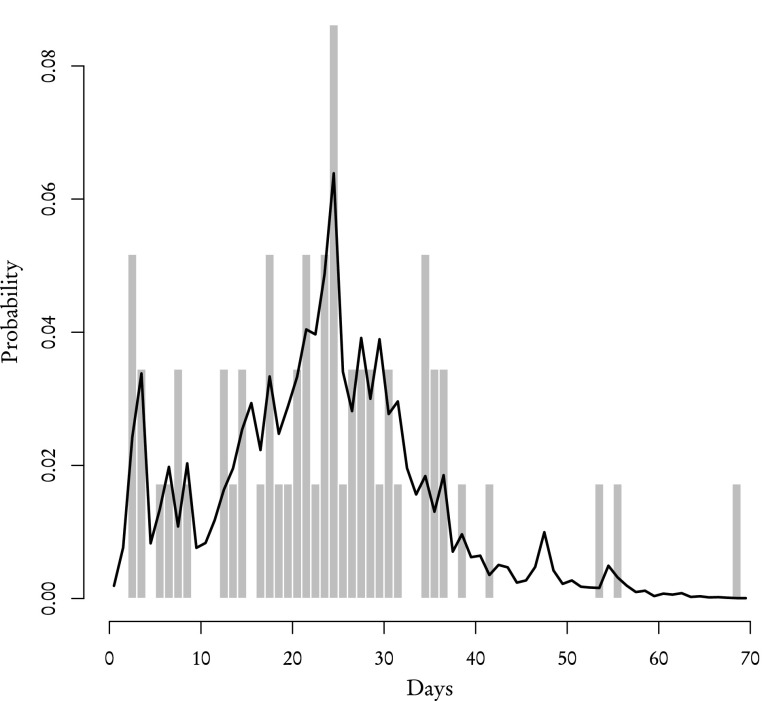
The probability distribution of review time for **known** reviewers: theoretical—*black line*, from data—*grey bars*

**Fig. 7:**
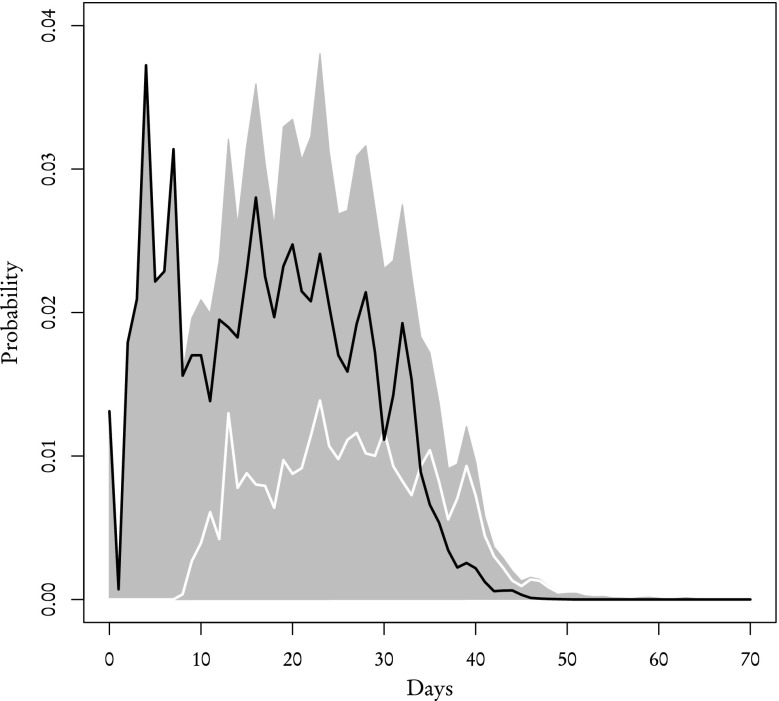
The theoretical probability distribution of review time for **other** reviewers who responded to the initial invitation (*black line*), who received an inquiry (*white line*) and their sum which gives the distribution for all **other** reviewers (*filled polygon*)

**Fig. 8:**
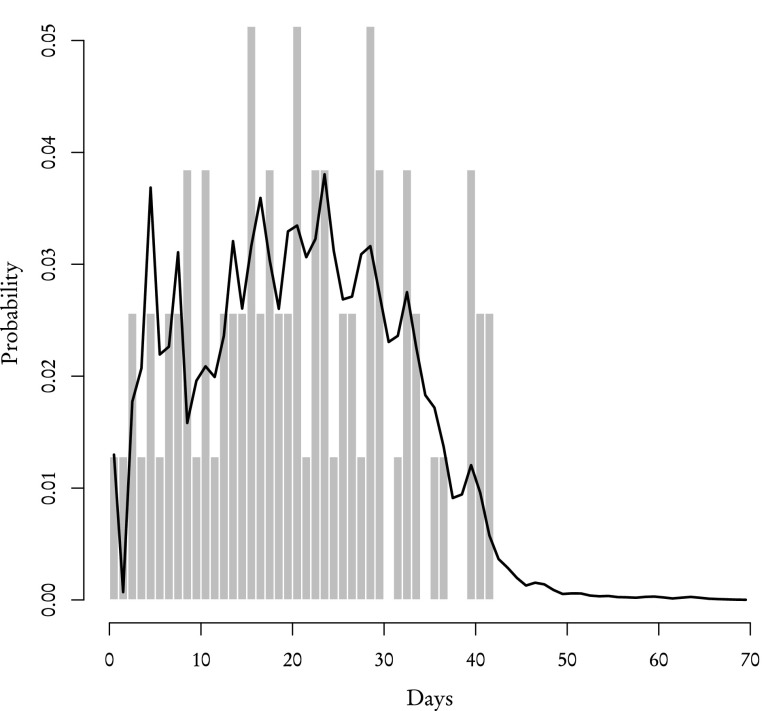
The probability distribution of review time for **other** reviewers: theoretical—*black line*, from data—*grey bars*

Using the aforementioned procedure we recreated the distribution of review times for both **known** and **other** reviewers which we then compared with the corresponding empirical distributions from the sample (Figs. 5, 6, 7 and 8). According to our theoretical calculations based on Eqs. (1–4) the average review time for **known** reviewers is 23 days with standard deviation of 12 days which is in agreement with the average review time acquired from the sample. As for **other** reviewers, the theoretical average review time is 20 days with standard deviation of 11 days and the sample, again, yields the same values. One-sample Kolmogorov–Smirnov test performed to compare the theoretical distribution with the sample gives *p* value 0.88 for **known** reviewers and 0.97 for **other** reviewers. It means that the distributions of review times calculated using partial distributions are essentially the same as the ones obtained directly from data.

This is an important and non-obvious observation, as the only underlying assumption behind Eqs. (1–4) is that the review process is memoryless (Markovian)—that is the partial distributions assigned to edges do not depend on the history of the process. Results presented thus far seem to confirm this reasonable assumption. Moreover, the findings are reinforced even further in the following section through simulations of the model.

Other than the validity of theoretical distributions, there are two main conclusions that can be drawn from results presented in Figs. 5, 6, 7 and 8. Firstly, the review time distribution is bimodal. Reviewers who either confirmed or sent in their reviews after receiving the invitation are the ones who contribute to the leftmost maximum (and they are in the majority of those who actually completed the reports—69 % of **other** and 82 % of **known**). Secondly, distributions of review time are similar for **known** or **other** reviewers. The difference between means and standard deviations for both groups is negligible from any practical standpoint: a two-sample Kolmogorov–Smirnov test for both empirical distributions gives *p* value $\simeq$0.40. Based on these facts one can make a very strong assumption that the distribution of review time is the same across the entire population of reviewers and does not depend on the reviewer group.

While in our work we were mostly interested in the time that is needed to acquire a given number of reviews, it should be mentioned that technically this is only the first major stage of the full peer review process. The second stage begins when the reviews are sent to authors and ends with the notification of acceptance or rejection. However, the dynamics of that second stage are rather linear and straightforward. In the case of our data from JSCS, one revision of the original manuscript was necessary to address the remarks of reviewers (though one has to keep in mind that we only had access to data pertaining to accepted manuscripts). On average, it took authors 34 days to deliver the revised version and final notifications were sent after 8 more days. Thus, manuscripts were accepted on average 42 days after the sub-editor received all reviews. It means that the second stage of the peer review process is longer than the first one, which is consistent with findings of other researchers (Trimble and Ceja 2011).

## Simulations of the review process

So far we have considered review times of a single reviewer. However, editors usually need more than one review in order to judge whether to publish an article. In the case of our data from JSCS, the sub-editor aims for two reviews per article and sent invitations to five reviewers on average—one **known** and four **other**. While this review strategy indeed resulted in two reviews per article on average (2.34 to be exact), 9 articles were published after receiving only one review, 24 after 2 reviews, 21 after 3 and 4 after 4 reviews. This discrepancy between the target number of reviews and the number of reviews actually received stems from the difference in the probability of finishing the report between **known** and **other** reviewers. We are going to call this probability the *completion rate*.

**Fig. 9:**
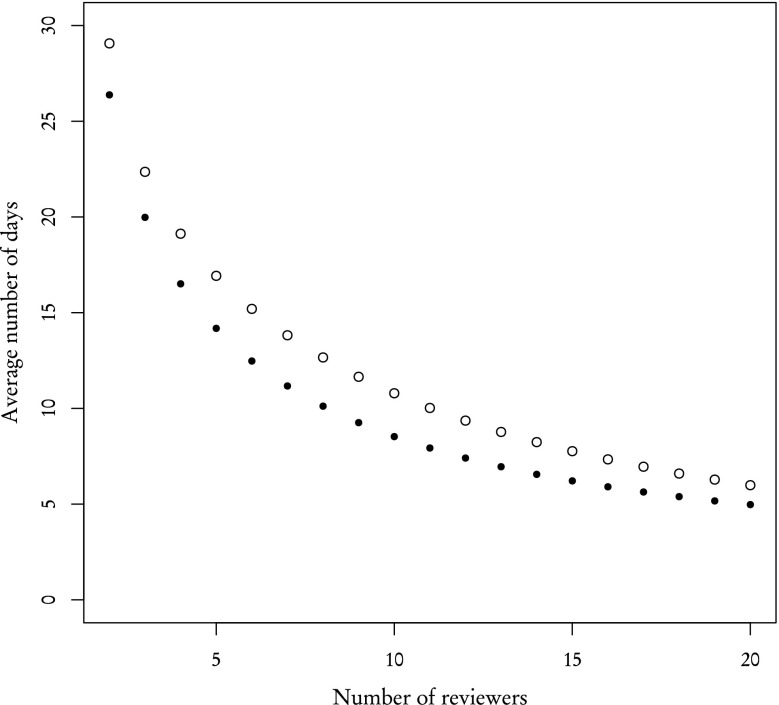
Average time of acquiring two reviews for **known** (*empty circles*) and **other** (*filled black circles*) reviewers when all reviewer finish their reviews

Using partial distributions we can easily simulate the effects of any editorial strategy and find the number of reviewers needed to achieve a certain number of reviews per article. We will use the average time of receiving two reviews as a measure of the effectiveness of each strategy. Figure 9 shows these average times under the assumption that the invited reviewer always writes the report (the completion rate equals 1 for both **known** and **other** reviewers) as a function of the number of reviewers. The average time decreases as the number of reviewers increases. Results for **known** and **other** reviewers are found to be very similar. This is intuitive and consistent with our prediction made in "Review time" section.

The assumption that an invitation always results in a report is not realistic. If we want to take into account the fact that the actual completion rate of the review process for a single reviewer is smaller than 1, especially for **other** reviewers, then some additional strategy needs to be introduced to deal with the cases when two reviews are not received at all. In our simulations, we decided to use a simple strategy: if two reviews are not received, then invitations are resent to the same number of reviewers. This procedure is repeated if necessary until reviewers produce two reports in total. While this is not the most effective and time-efficient strategy which we would suggest to editors, it still allows us to study the consequences of the difference between the completion rates of **known** and **other** reviewers.

**Fig. 10:**
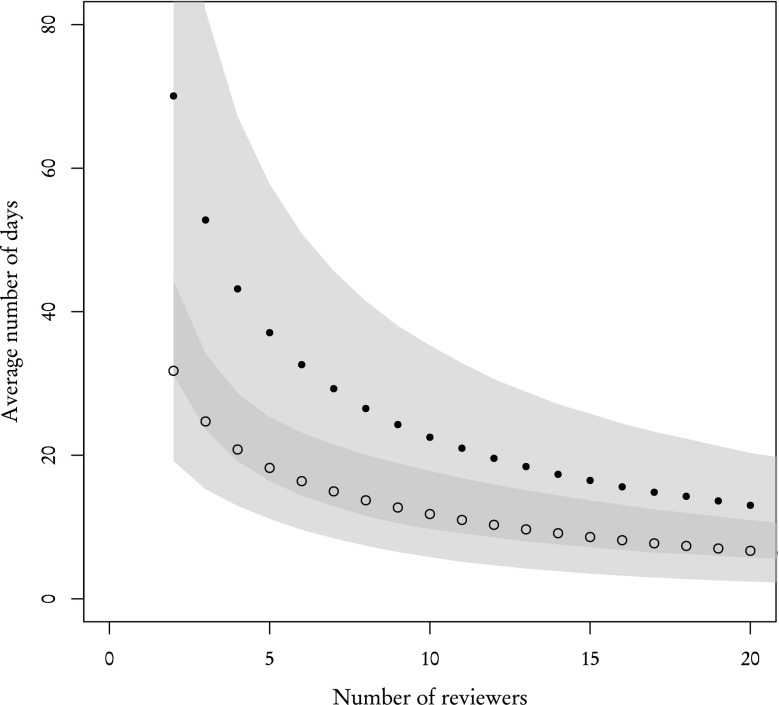
Average time of acquiring two reviews for **known** (*empty circles*) and **other** (*filled black circles*) reviewers with completion rate taken into account. *Filled polygon* represents standard deviation

Figure 10 is analogous to Fig. 9—in that it shows the average time of receiving two reviews—but this time we used the actual completion rates taken from the sample (89 % for **known**, 31 % for **other** reviewers) and employed the policy described in the previous paragraph. As can be clearly seen, the difference in completion rates between **known** and **other** reviewers results in a completely different dynamics. **Other** reviewers are far less effective. Their average review time is much higher: for example, two reviews can be received from 2 **known** reviewers after 32 days, but **other** reviewers finish the set of 2 reviews after 70 days. Even as the number of reviewers increases, this difference remains significant.

**Fig. 11:**
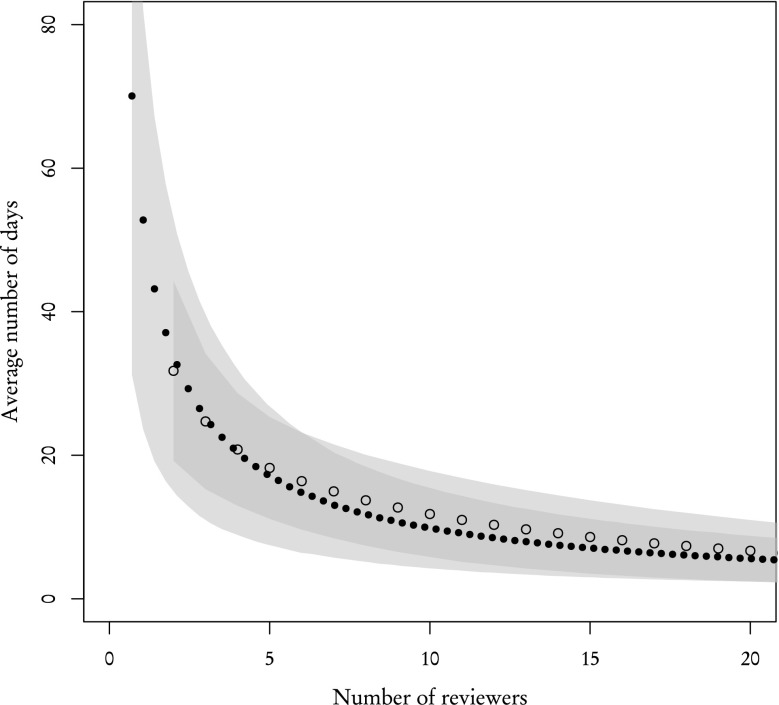
Same as Fig. 10 but with the X axis rescaled for **other** reviewers

However, in "Review time" section, we have shown that distributions of review time for **known** and **other** reviewers are very similar, which suggests that the completion rate is the leading factor during the review process. This claim is partially supported by results presented in Fig. 9. If that claim is indeed valid, then one **known** reviewer should be ”worth” 89/31 % **other** reviewers and conversely one **other** reviewer is ”worth” 31/89 % **known** reviewers. By ”worth”, we mean that proportionally substituting one type of reviewer for another should yield the same results. Figure 11, where the X axis for one type of reviewers was rescaled to match their worth in the other type of reviewers, confirms this prediction. The average number of days after which 2 reviews are acquired are similar and standard deviations, while not exactly the same—which is to be expected are comparable.

**Fig. 12:**
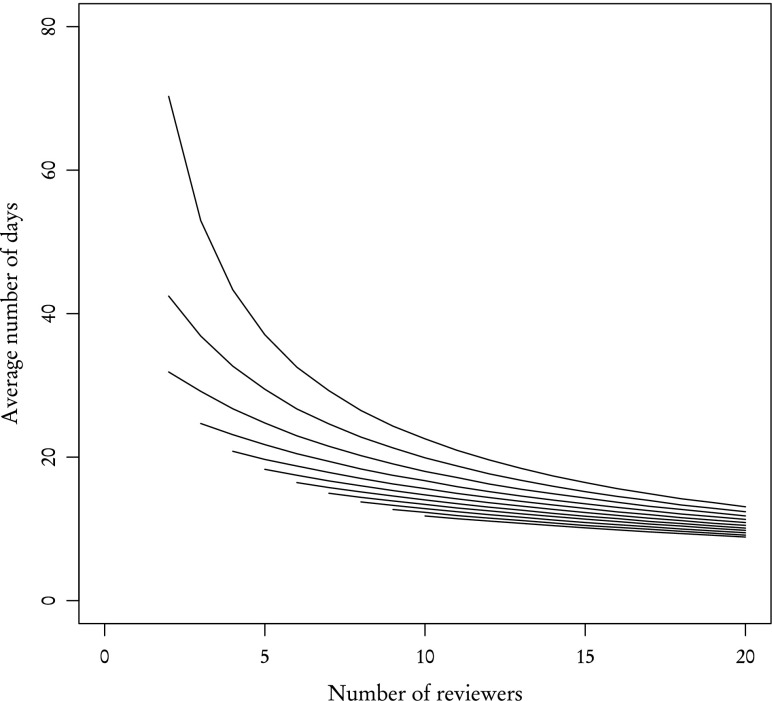
Average time of acquiring two reviews for a group of mixed reviewers. The X axis—total number of reviewers. *Curves* correspond to various numbers of **known** reviewers: 0 **known**—*top curve*, 10 **known**—*bottom curve*

**Fig. 13:**
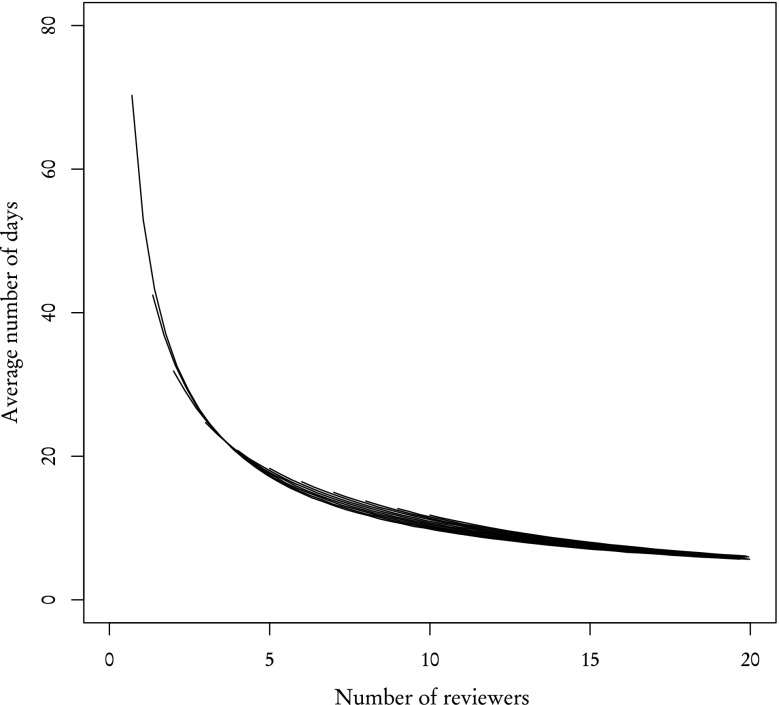
Same as Fig. 12 but with rescaled X axis

So far we have studied separately **known** and **other** reviewers. However, as explained in "Review process and initial data analysis" section, the group of reviewers invited to review an article usually contains reviewers of both kinds. Figure 12 shows the average time of acquiring two reviews when reviewer types are mixed with different proportions. As one could expect, the average time decreases with the increasing total number of reviewers and **known** reviewers are far more effective than **other**. Still, by rescaling the X axis—that is by expressing the worth of one kind of reviewer using another—we get similar results (Fig. 13).

**Fig. 14:**
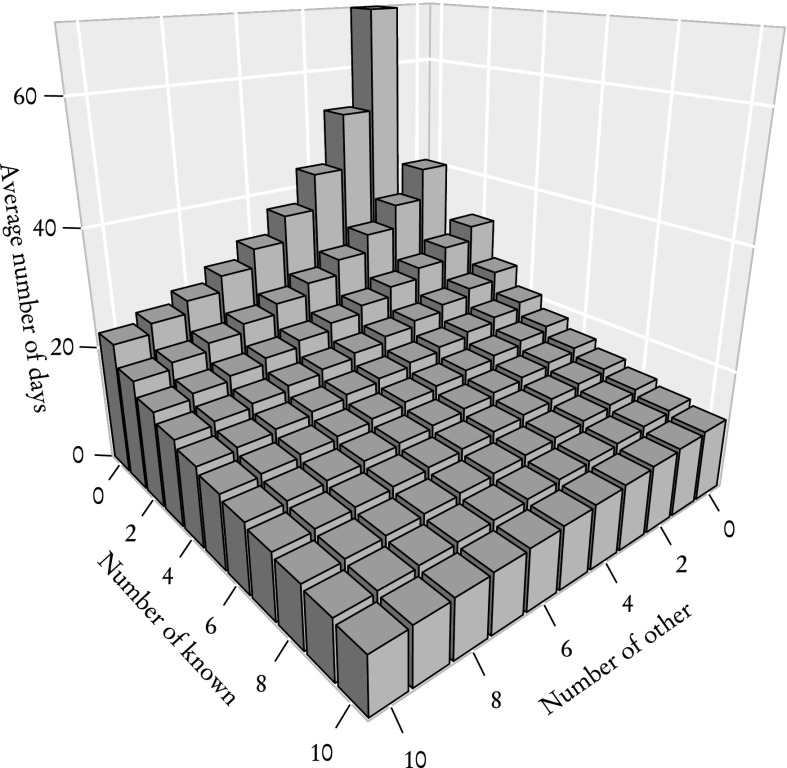
Average time of acquiring two reviews for a group of mixed reviewers

Information about average times in groups of mixed reviewers, expressed in a slightly different way in Fig. 14 and summarised in Table 1 can potentially be of great importance for editors and act as a guide in determining the optimal number of reviewers. For example, in order to receive two reviews after about 30 days, one needs to invite 7 **other** reviewers, 2 **known** or a mixed group of 4 **other** and 1 **known**. That last option is consistent with the choice made by the sub-editor of JSCS who provided us with the data.

**Table 1:** Average number of days needed to receive two reviews from a group of reviewers with a given number of **known** (columns) and **other** (rows) reviewers

$N_o$	$N_k$									
	0	1	2	3	4	5	6	7	8	9
0	–	–	32	25	21	18	16	15	14	13
1	–	42	29	23	20	17	16	14	13	12
2	70	37	27	22	19	17	15	14	13	12
3	53	33	25	20	18	16	15	13	12	11
4	43	29	23	19	17	15	14	13	12	11
5	37	27	22	18	16	15	14	13	12	11
6	33	25	20	17	16	14	13	12	11	10
7	29	23	19	17	15	14	13	12	11	10
8	26	21	18	16	14	13	12	11	11	10
9	24	20	17	15	14	13	12	11	10	10

Values for groups of reviewers smaller than two were omitted

It is important to note that editors may be tempted to invite only **known** reviewers, which would lead to shorter review times. However, such a policy would not only be unrealistic but also inadvisable. The pool of potential **known** reviewers is limited and editors would be forced to invite the same reviewers several times within a short time frame. This, in turn, could discourage such reviewers and make them more likely to turn down invitations, further reducing the pool. It gives us an idea that the process of selecting reviewers could be modeled as an optimization problem within an agent-based simulation framework (where other factors, e.g. the quality of reviewers (Ausloos et al. 2015), could be taken into account), however we leave it to further studies.

## Discussion with conclusion

In summary, we have examined selected aspects of peer review through a case study—an analysis of the review stages of 58 papers submitted to the Biochemistry and Biotechnology section of the Journal of the Serbian Chemical Society. While it would be interesting to compare these results with those obtained by studying other journals, such data is not easily available. On one hand, large publishers treat such information as trade secrets and are not willing to share it with external researchers. On the other hand, smaller publishers often do not have the IT infrastructure that would allow for automatic data retrieval (e.g. submissions are via e-mail only) and data must be collected manually by editors, which is a very time consuming process. However, during the last PEERE EU project workshop on ”New models of peer review” (November, 2015) where we presented our findings, we were approached by editors willing to give us access to larger collections of data. Thus, we may be able to provide some comparative analysis in the future.

We have studied review time that characterises the entire process as well as the durations of all stages. We have used a directed graph to describe the process and found empirical weights that correspond to the probability of passing through each edge. We have introduced two kinds of reviewers—**known** and **other**—and found that

- the distribution of review time is similar for both kinds of reviewers
- but the completion rate is much higher for **known** reviewers than for **other** reviewers.

Therefore, the completion rate is the main factor that determines the effectiveness of the review process.

We have simulated the editorial workflow using a Markov-like model. We have tested the impact of some modifications of the editorial policy on the efficiency of the whole review process, emphasizing the number of different types of reviewers in particular. Our results suggest that **known** reviewers are objectively better than **other** reviewers and there is no advantage in choosing the latter over the former. In an ideal world, editors should invite only **known** reviewers. Unfortunately, since they are effectively a finite resource, this is not possible.

In our opinion, the difference between the completion rate of **known** and **other** reviewers can be explained in two ways:

- It is a purely statistical effect. It is possible that the completion rate of **other** reviewers is a good estimate of the average completion rate of the entire population, but by virtue of sheer luck the sub-editor knows only reviewers with high completion rate, who belong to the tail of the distribution. On the other hand, the opposite is also possible—the completion rate of the entire population is high, but the sub-editor chooses **other** reviewers from the tail of the distribution.
- The relationship with the editor—the fact that some reviewers know the editor personally—determines the completion rate of **known** reviewers.

We believe that the first explanation is highly unlikely and that the second one is correct. Reviewers have only finite amounts of time at their disposal. Usually, they cannot accept all invitations they receive which forces them to make a choice. It seems intuitive that reviewers will prioritise invitations from editors they know in order to maintain reputation and avoid disappointing these editors. In fact, according to the JSCS sub-editor, not only are the **known** reviewers more likely to accept the invitation than **other** reviewers, but they are also more diligent and write reviews of high quality. However, in the absence of such a personal relationship with the editor, reviewers may employ different criteria. For example, it is common sense that in general reviewers will choose more prestigious journals with high impact factor over the less prestigious ones.

Based on the aforementioned observations, we would like to propose a hypothesis that the completion rate is not necessarily a property of reviewers but of their relationships with other entities—be it journals, editors or even other reviewers. As such, the same reviewer can be treated as reliable by some journals or journal editors—i.e. likely to answer and write the review—and as unreliable by others. Editors, at least very roughly, should be able to estimate the completion rate of potential reviewers. For example, a journal with low impact factor cannot feasibly expect a review from a Nobel laureate. Moreover, since relations between people can change, the completion rate does not have to be constant and it may evolve with time.

Authors of manuscripts, reviewers and editors form a complex network of mutual connections, the structure of which has a direct influence on the effectiveness of the review process. However, since editors are the ones who actually manage the entire process, it would seem that their workflow is equally, if not even more important than that structure. With the right kind of workflow, one can potentially overcome many shortcomings of the behaviour of both authors and reviewers. We have shown that through very naive and most certainly not optimal means—by sending invitations to a sufficiently large group of potential reviewers—it is possible to achieve short review time. The results presented in this manuscript can be used as a foundation necessary to study the dynamics of the review process and determine the optimal workflow for an editor, which can be the subject of interesting research work.
